# Genome-Wide Analysis Reveals Genetic Potential for Aromatic Compounds Biodegradation of *Sphingopyxis*

**DOI:** 10.1155/2020/5849123

**Published:** 2020-05-27

**Authors:** Fei Yang, Hai Feng, Isaac Yaw Massey, Feiyu Huang, Jian Guo, Xian Zhang

**Affiliations:** ^1^Department of Occupational and Environmental Health, Xiangya School of Public Health, Central South University, Changsha, China; ^2^Hunan Provincial Key Laboratory of Clinical Epidemiology, Central South University, Changsha, China

## Abstract

Members of genus *Sphingopyxis* are frequently found in diverse eco-environments worldwide and have been traditionally considered to play vital roles in the degradation of aromatic compounds. Over recent decades, many aromatic-degrading *Sphingopyxis* strains have been isolated and recorded, but little is known about their genetic nature related to aromatic compounds biodegradation. In this study, bacterial genomes of 19 *Sphingopyxis* strains were used for comparative analyses. Phylogeny showed an ambiguous relatedness between bacterial strains and their habitat specificity, while clustering based on Cluster of Orthologous Groups suggested the potential link of functional profile with substrate-specific traits. Pan-genome analysis revealed that 19 individuals were predicted to share 1,066 orthologous genes, indicating a high genetic homogeneity among *Sphingopyxis* strains. Notably, KEGG Automatic Annotation Server results suggested that most genes pertaining aromatic compounds biodegradation were predicted to be involved in benzoate, phenylalanine, and aminobenzoate metabolism. Among them, *β*-ketoadipate biodegradation might be the main pathway in *Sphingopyxis* strains. Further inspection showed that a number of mobile genetic elements varied in *Sphingopyxis* genomes, and plasmid-mediated gene transfer coupled with prophage- and transposon-mediated rearrangements might play prominent roles in the evolution of bacterial genomes. Collectively, our findings presented that *Sphingopyxis* isolates might be the promising candidates for biodegradation of aromatic compounds in pollution sites.

## 1. Introduction

Biodegradation of hazardous pollutants mediated by microorganisms is widely regarded as an effective strategy for reducing the risk of toxins [1]. Commonly, aromatic compounds are organic molecules that contain one or more aromatic rings, especially benzene ring, and are the most concerned environmental pollutants that severely threaten the environment and human health due to their prevalent and persistent characteristics and bioaccumulation via food web [2]. Over decades, various bacteria, such as *Pseudomonas*, *Burkholderiales*, and *Rhodococcus* [3–5], have been identified to harbor the ability of individually utilizing aromatic compounds as alternative carbon and energy source under the condition of nutrient-deficiency. On the basis of phylogenetic, chemotaxonomic, and physiological analyses, Takeuchi and Maruyama have stated that genus *Sphingomonas* were redivided into five independent genera (i.e., *Sphingomonas*, *Sphingopyxis*, *Sphingobium*, *Novosphingobium*, and *Sphingosinicella*) [6]. To our knowledge, strains of *Sphingobium*, *Sphingomonas*, and *Novosphingobium* have been extensively studied with respect to their potential for aromatic compounds degradation [7, 8].

In various habitats, members of *Sphingopyxis* isolates have been reported to efficiently degrade the aromatic compounds, such as microcystins (MCs) [9], tetralin [10], styrene [11], and triclosan [12], which generally cause environmental pollution and induce negative impact on human and ecosystem health [13]. For instance, *Sphingopyxis* sp. C-1 isolated from an eutrophic lake in China has been shown to harbor the ability of MCs degradation [9]. *Sphingopyxis fribergensis* Kp5.2, originally isolated from soils, was reported to degrade styrene [14]. In addition, *Sphingopyxis granuli* TFA isolated from the Rhine river was able to grow in organic solvent tetralin [15]. However, not all isolates exhibited the capability of aromatic compounds degradation. For example, *Sphingopyxis ummariensis* UI2, *Sphingopyxis indica* DS15, and *Sphingopyxis flava* R11H were isolated from different hexachlorocyclohexane (HCH)-contaminated soils, but there was no evidence to support their biodegradation capabilities. Furthermore, it was unclear whether *Sphingopyxis* sp. MWB1 had the ability of crude-oil-degradation, although it was isolated from crude oil-contaminated seashore [16]. There is a functional diversity among *Sphingopyxis* strains; it is thus of interest to explore the genetic potential for cleanup of pollutants.

Currently, a strain YF1 was isolated from the eutrophic Lake Taihu and phylogenetically affiliated to genus *Sphingopyxis* [17]. In our study, 18 reference genomes of other *Sphingopyxis* isolates were selected for comparison. Phylogeny of *Sphingopyxis* strains was inferred based on multiple methods to verify their evolutionary relationships. Comparative genomics was further performed to systematically analyze the functional profile and metabolic potential of *Sphingopyxis* isolates to investigate their genetic potential for aromatic compounds biodegradation.

## 2. Material and Methods

### 2.1. Strains Selection in This Study

In this study, 16S rRNA-based phylogeny showed that strain YF1 was phylogenetically affiliated to genus *Sphingopyxis* (Figure S1). Subsequently, 18 other *Sphingopyxis* strains that were originally isolated from various ecological niches, such as contaminated soil, marine water, and eutrophic lake, were used for further analysis. The general feature for each *Sphingopyxis* isolate was summarized in Table 1.

### 2.2. Bacterial Phylogenetic Analysis

We employed four distinct methods for phylogenetic analysis to confirm the phylogenetic relationships among 19 *Sphingopyxis* strains. Multiple-sequence alignment of 16S rRNA genes was performed using ClustalW [18], and the phylogenetic tree was then constructed using MEGA version 7.0 [19] with the neighbor-joining method. Herein, the evolutionary distance was calculated with 1,000 bootstrap replicates. In order to ensure the reliability of the subsequent analysis, genome sequences of 19 *Sphingopyxis* strains were assessed using BUSCO v3 based on evolutionarily informed expectations of gene content with a proteobacteria_odb9 BUSCO lineage datasets containing 221 BUSCOs [20, 21]. Orthologous groups of proteins were identified amongst 19 *Sphingopyxis* strains using OrthoFinde [22] with diamond search and Markov Cluster Algorithm. All single-copy genes in 19 strains were aligned using MAFFT [23], and phylogenetic analysis was performed using MEGA version 7.0. Topology for species tree was constructed using CVTree3 [24] with *K*-tuple length of 6. Furthermore, values of average nucleotide identity (ANI) between pairs of *Sphingopyxis* genomes were calculated using web server JspeciesWS [25].

### 2.3. Orthologous Proteins Identification and Functional Annotation

All-versus-all BLASTP with an *E* value cut-off of 0.00001 was performed using the extracted protein sequences from each *Sphingopyxis* strain. The output results were used for further analysis, as described in previous studies [26–29]. Bacterial Pan Genome Analysis (sequence identity ≥50%; and *E* value ≤1e^−5^) was applied for the identification of orthologous genes shared by 19 *Sphingopyxis* strains [30]. The orthologous groups were classified as shared, distributed, and/or unique genes according to their distribution across bacterial genomes. Furthermore, the extracted sequences of shared and distributed genes were assigned into Clusters of Orthologous Groups (COG) categories by aligning against eggNOG v4.5.1 using the eggNOG-mapper tool [31]. Also, possible genes in all *Sphingopyxis* genomes were annotated using the KEGG Automatic Annotation Server [32]. Notably, the KEGG database [33, 34] was used for the identification of KEGG Orthology (KO), and genes potentially related to aromatic compounds metabolism were screened according to KO annotation. Finally, all results were manually checked.

### 2.4. Prediction of Mobile Genetic Elements (MGEs)

In order to investigate the evolution of *Sphingopyxis* strains, MGEs were detected in this study. Genomic islands (GIs) of *Sphingopyxis* spp. were predicted using the web server IslandViewer 4 [35] with methods SIGI-HMM [36] and IslandPath-DIMOB [37]. Insertion sequences (ISs) were identified by BLAST comparison (*E* value ≤1e^−5^) against the ISFinder database [38]. Online server CRISPRFinder [39] was employed to identify the Clustered Regularly Interspaced Short Palindromic Repeats (CRISPR) arrays via BLAST search against dbCRISPR (CRISPR database). In many bacterial and archaeal genomes, CRISPR were generally considered to be important for prokaryotic immunity to resist the phages and plasmids [26]. In addition, prophage sequences were detected using PHAST (PHAgeSearch Tool) [40] with default parameters.

## 3. Results and Discussion

### 3.1. *General Features of 19* Sphingopyxis *Gnomes*

The general features of 19 *Sphingopyxis* strains are summarized in Table 1. Among these strains, *S. macrogoltabida* 203N and *S. fribergensis* Kp52 isolated from contaminated soil were identified to harbor a larger genome of over 5 Mbp, and *S. baekryungensis* DSM 16222 isolated from marine water had the smallest genome (3.07 Mbp). Except for YF1 and DSM 16222, all *Sphingopyxis* strains contained one copy of 16S rRNA gene. The small 16S rRNA copy number is a characteristic commonly associated with bacteria that inhabit the specialized environments and slowly respond to changing habitat conditions [41]. In addition, strain YF1 with a chromosome of 4.37 Mbp had the highest GC content of 66.6% compared to other *Sphingopyxis* strains.

### 3.2. *Phylogenetic Analyses of* Sphingopyxis *Strains*

**Figure 1:**
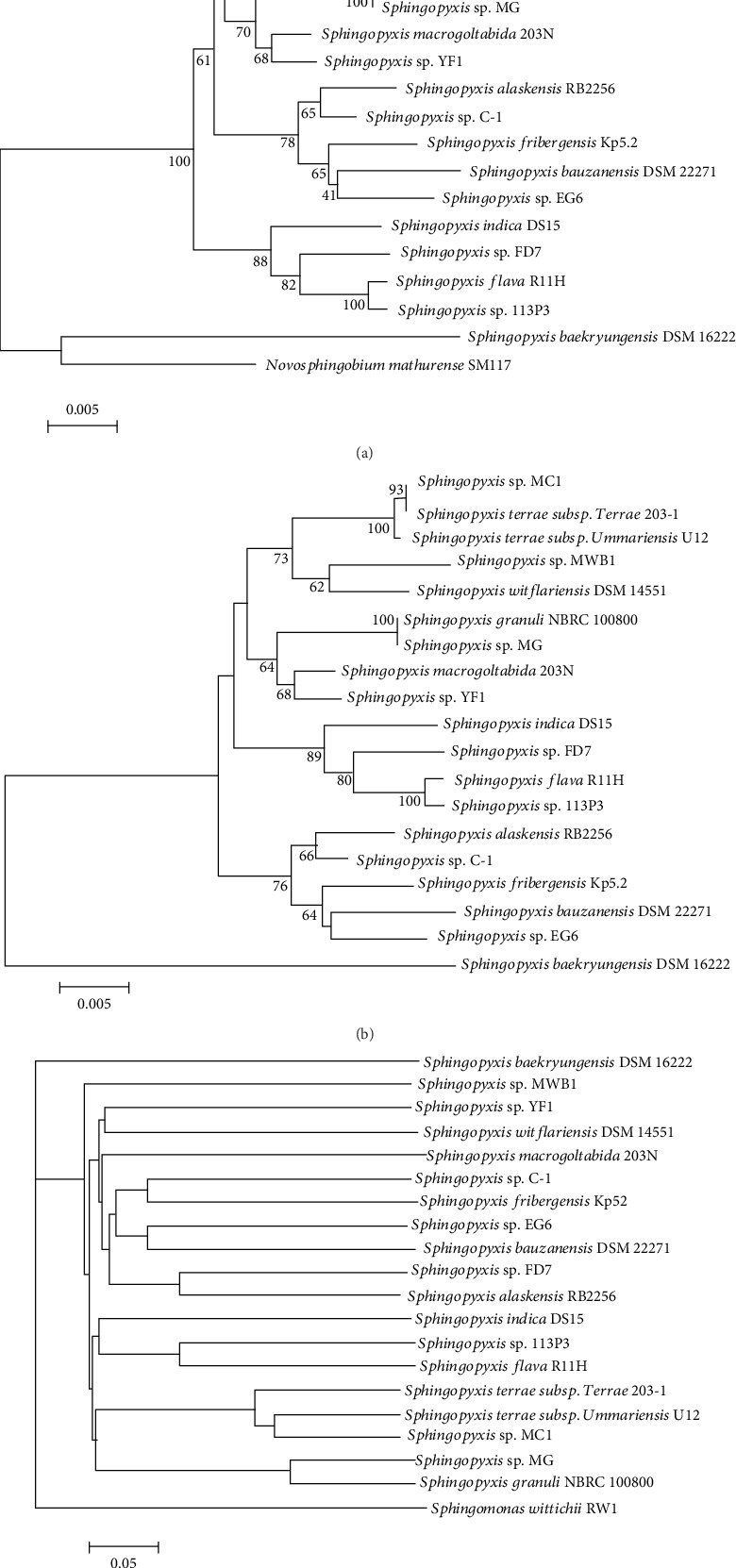
Phylogeny of 19 *Sphingopyxis* strains. Phylogenetic trees based on (a) 16S rRNA genes and (b) 1334 single-copy genes were constructed. The bars represent the number of substitutions per nucleotide position. Percentage bootstrap values (≥50%) were shown next to the nodes. *Novosphingobium mathurense* SM117 was used as an outgroup. (c) Whole-genome-based phylogenetic tree was generated using a composition vector approach with *K*-tuple length of 6. *Sphingomonas wittichii* RW1was set as an outgroup.

Genome completeness of 19 *Sphingopyxis* strains was evaluated, and all values were higher than 92%, which ensured the reliability of the subsequent analysis. Phylogenetic relationships revealed that strain YF1 had an ambiguous status based on four different phylogenetic analyses (Figure 1 and Table S1). For instance, both 16S rRNA genes fragment and single-copy genes phylogenetic tree showed that strain YF1 was likely to be assigned into *S. macrogoltabida* species, while whole-genome-based phylogeny provided evidence that strain YF1 had a close relationship with *S. witflariensis* DSM 14551. In our study, ANI values between pairs of *Sphingopyxis* isolates were further calculated (Table S1), and the results (<95%) suggested that strain YF1 might be phylogenetically affiliated to a novel species of genus *Sphingopyxis*. Furthermore, phylogenetic analyses based on four different methods revealed that *Sphingopyxis* sp. MC1, *Sphingopyxis terrae* subsp. *terrae* 203-1, and *Sphingopyxis terrae* subsp. *Ummariensis* UI2 were grouped together in a separate clade, suggesting that strain MC1 might belong to *Sphingopyxis terrae* species. Similarly, *Sphingopyxis* sp. MG and *S. granuli* NBRC 100800 were clustered into a distinct group, and ANIb value (96.8%) further supports the phylogenetic relationship. In addition, *Sphingopyxis* sp. 113P3 and *S. flava* R11H, as well as *Sphingopyxis* sp. EG6 and *S. bauzanensis* DSM 22271 were grouped together, but ANI values further determined their taxonomy as separate species. Notably, strain DSM 16222 (formerly belonging to *S. baekryungensis*) was assigned into an outgroup, which suggested that this strain was likely to be phylogenetically affiliated to another genus instead of *Sphingopyxis* genus.

### 3.3. *Pan-genome Analysis Reveals Difference in Gene Repertoire of* Sphingopyxis

**Figure 2:**
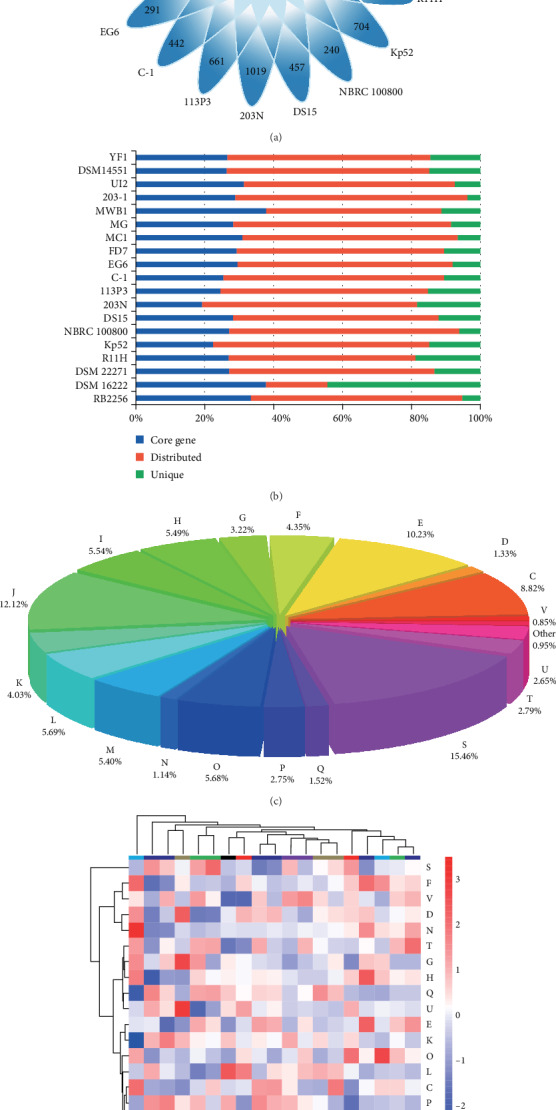
Comparison of orthologous groups in 19 *Sphingopyxis* genomes. (a) Venn diagram showing the numbers of shared genes and flexible genes in each *Sphingopyxis* strains. (b) Percentages of shared, distributed, and unique genes in each of the19 *Sphingopyxis* genomes. (c) Functional assignment of core-genome shared by 19 *Sphingopyxis* strains. (d) Functional profiling of the 19 *Sphingopyxis* genomes. Heatmap indicated the normalized relative abundance of COG categories of protein-coding genes in each *Sphingopyxis* genomes. Strains and COG categories were clustered using the Euclidean distance. The color scale represented the relative abundance of each COG category, normalized by sample mean. Abbreviations: C: energy production and conversion; D: cell cycle control; E: amino acid transport and metabolism; F: nucleotide transport and metabolism; G: carbohydrate transport and metabolism; H: coenzyme transport and metabolism; I: lipid transport and metabolism; J: translation, ribosomal structure, and biogenesis; K: transcription; L: replication, recombination, and repair; M: cell wall/membrane/envelope biogenesis; N: cell motility; O: posttranslational modification, protein turnover; P: inorganic ion transport and metabolism; Q: secondary metabolites biosynthesis and transport; S: function unknown; T: signal transduction mechanisms; U: intracellular trafficking, secretion, and vesicular transport; V: defense mechanisms.

In this study, 68,244 protein-coding genes (CDS) from *Sphingopyxis* strains were clustered into 6,438 orthogroups. The number of orthogroups in these strains ranged from 2,477 to 5,003, indicating that most genes had no multiple copies. Comparative analysis further revealed that the percentage of shared genes (1,066) in each *Sphingopyxis* genome varied from 23.92% to 38.30% (Figure 2(a)-2(b)). This was similar to a previous study, in which *S. granuli* TFA harbored 1,371 shared genes in its gene repertoire [42]. In contrast, there was a relatively low percentage of core genome shared by *Sphingomonas*, *Sphingobium*, and *Novosphingobium* members. For example, comparative analyses based on 6, 22, and 27 *Novosphingobium* strains showed that 929, 674, and 220 shared genes were identified, respectively [43–45]. Another comparative genomics further suggested that 492 CDS were conserved in the complete genomes, and 268 CDS were universally conserved in all genomes of 26 bacterial strains, including 13 *Sphingomonas* spp., six *Sphingobium* spp., six *Novosphingobium* spp., and one *Sphingopyxis* sp. [7]. The findings indicated that genus *Sphingopyxis* was more conservative than its neighboring genera such as *Novosphingobium* and *Sphingomonas* with respect to their gene content [46]. In other words, core-genome and pan-genome had a pronounced heterogeneity in these neighboring genera, which suggested that caution should be taken when scaling from a single genus to the larger neighboring genera. Furthermore, the percentage of unique genes in *S. baekryungensis* DSM 16222 (44.99%) was much higher than that in other strains (3.97-20.23%). The result, to some extent, also supported that *Sphingopyxis* genus was a compact group, and the affiliation of strain DSM 16222 should be revised [42]. In addition, up to 20%, unique genes were predicted to be present in *S. macrogoltabida* 203N as its largest genome size contains a chromosome and two plasmids. With the exception of strain DSM 16222, the percentages of distributed genes in the 18 *Sphingopyxis* genomes varied from 52.17% (strain R11H) to 65.57% (strain YF1), and the proportions were relatively higher than that in previous studies [45]. In general, the high proportion of distributed genes were not essential to basic lifestyle, but they might confer bacteria with special features such as niche adaptation and reflect their variable metabolic profiles.

COG assignment was performed to categorize the function of gene families in *Sphingopyxis* core-genome (Figure 2(c)). Results showed that a large number of CDSs were assigned into COG category [S] (function unknown, 16%). In addition, numerous genes were matched to COG categories [J] (ribosomal structure and biogenesis), [E] (amino acid transport and metabolism), and [C] (energy production and conversion), which accounted for 31.23% of the total shared genes. These essential genes were related to gain and loss of genetic information, uptake of nutrients from various environments, as well as the sustainment of basic lifestyle. Similarly, KO annotation showed that most of the shared genes in *Sphingopyxis* strains were involved in genetic information processing. Furthermore, there were many genes associated with carbohydrate and amino acid metabolism. With respect to pan-genome of 19 *Sphingopyxis* strains, the four most abundant CDSs were classified into COG categories [K] (transcription), [E], [P] (inorganic ion transport and metabolism), and [C]. In addition, functional analysis revealed that strain-specific genes were assigned into different COG categories in individuals, and their abundances were diverse.

Microorganisms could rearrange their metabolic profiles to better adapt to specific habitats and utilize the compounds to which they were exposed in eco-environments. In this study, COG clustering suggested a potential correlation between bacterial strains with their habitat specificity (Figure 2(d)). For instance, strains YF1 and C-1 originally isolated from cyanobacterial blooms were grouped together in the functional heat map. Similarly, *S. bauzanensis* DSM 22271 and *S. flava* R11H from hydrocarbon-contaminated soil were clustered into one subgroup, and *S. fribergensis* Kp52 from meadows, which were noncontaminated or contaminated with aliphatic and aromatic hydrocarbons, was clustered with *S. indica* DS15 that was from an HCH-contain dumpsite.

### 3.4. Pathways Prediction for Aromatic Compounds Degradation

**Figure 3:**
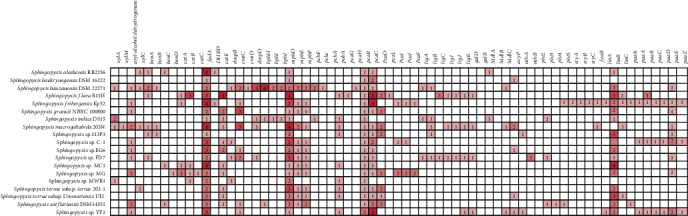
Comparison of main genes potentially involved in aromatic compounds degradation among *Sphingopyxis* strains. White box: absence of genes; red box: presence of genes with the number of copies in each strain. More details were listed in Table S2.

In order to understand the metabolic diversity among 19 *Sphingopyxis*, we assigned CDSs into the KEGG database to identify the functional genes potentially involved in metabolisms of aromatic compounds. KAAS results suggested that there were many genes/gene clusters related to aromatic compounds degradation in *Sphingopyxis* strains (Figure 3, Figure S2, and Table S2). *β*-ketoadipate pathway, the most widely used aromatic compound-degrading pathway in microorganisms [4], was predicted in *S. bauzanensis* DSM 22271, *S. flava* R11H, and *Sphingopyxis* sp. MG. Genomic regions containing *pca* genes related to protocatechuate metabolism and *cat* genes were present in *S. flava* R11H and *Sphingopyxis* sp. MG, while genes for catechol degradation were identified in *S. bauzanensis* DSM 22271. In *S. macrogoltabida* 203N and *S. bauzanensis* DSM 22271, *xyl* genes associated with the catalytic reaction of benzoate to generate key intermediate of catechol were identified. Furthermore, *bph* genes involved in the degradation of polycyclic aromatic hydrocarbons were found in *S. bauzanensis* DSM 22271 [47]. More specifically, *xyl* genes were found to be clustered with *bph* genes in *S. bauzanensis* DSM 22271, but no *cat* genes were found in their flank. Likewise, *cmt* genes related to the cleavage of 2,3-dihydroxy-p-cumate to generate 2-hydroxypentadienoate [48] were identified to be clustered with *xyl* genes in *S. macrogoltabida* 203N. In addition, the two-component protocatechuate 4,5-dioxygenase (*ligAB*) was likely to be commonly involved in the cleavage of protocatechuate in *Sphingopyxis*. The nearly complete HCH-degrading pathway was only identified in *S. fribergensis* Kp52. In most *Sphingopyxis* strains, genes *linA* and *linB* encoding the dehydrochlorinase and haloalkane dehalogenase, respectively, were present, but the others have not been detected [49]. Among 19 *Sphingopyxis* isolates, it was suspected that four members isolated from the HCH-containing dumpsite had the potential to degrade HCH, but the absence of *lin* gene cluster suggested that these strains might undergo the loss event of genes related to HCH-degrading pathway which have been acquired at an early stage [50]. Notably, a complete pathway for phenylacetyl-CoA degradation was only present in *Sphingopyxis* sp. Kp5.2, while gene *PaaK* for phenylacetate-CoA ligase was absent in MC-degrading bacteria *Sphingopyxis* sp. YF1 and C-1 [51]. As previously reported, a *sty* gene cluster was present in *Sphingopyxis* sp. Kp5.2, which had the ability to convert styrene into phenylacetic acid [15, 52].

### 3.5. MGEs and CRISPRs Analysis

In general, MGEs such as phages, transposable, and IS elements are considered to be important for the evolution of microorganisms. Hence, MGEs were identified and compared among these *Sphingopyxis* genomes (Table 2 and Table S3). In our study, many MGEs existed in *Sphingopyxis* strains, suggesting their high plasticity and rapid adaptation in diverse environments. Some IS elements were observed in genomes of *Sphingopyxis* sp. YF1 (29), *Sphingopyxis* sp. MWB1 (35), and *Sphingopyxis* sp. C-1 (57), while these elements in *S. bauzanensis* DSM22271 (349), *S. flava* R11H (347), and *S. witflariensis* DSM 14551 (345) were predicted to outnumber that in the formers. Further analysis suggested that most of IS elements were classified into the IS3 families. Analysis of genomic regions showed that several IS- and transposase-coding genes were predicted to be located at the up- and downstream of genes related to aromatic compounds degradation, indicating that these functional genes might be acquired via horizontal gene transfer (HGT) rather than vertical inheritance. More especially, *S. flava* R11H was predicted to harbor the IS6100 elements, which were reported as mosaic distribution in the neighborhood of *lin* genes for the HCH-degrading pathway [53, 54] and genes for carbazole conversion enzymes involved in carbazole degradation pathway [55]. However, no IS element was identified at the up- and downstream of *lin* genes in *S. fribergensis* Kp52, which indicated that these functional genes were likely to be gained via vertically inheriting. Notably, gene cluster associated with MCs degradation was identified to be located at a genomic island with GC content of 59.1%, which suggested that HGT events might occur during the evolution of MC-degrading genes.

Similar to *Novosphingobium*, *Sphingopyxis* strains were predicted to have few CRISPRs. In our study, two CRISPRs were identified in *S. witflariensis* DSM 14551 and *Sphingopyxis* sp. FD7. In addition, only one CRISPR was predicted to be present in *S. granuli* NBRC 100800 (with three spacers) and *S. indica* DS15 (with five spacers). As for other *Sphingopyxis* strains without CRISPRs, there might be a low frequency of viral attacks and vulnerability of the defense system.

## 4. Conclusions

**Figure 4:**
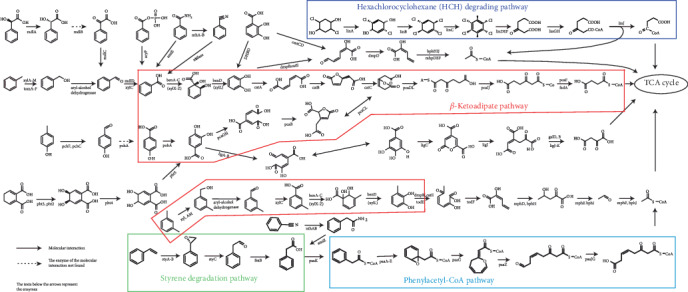
Prediction of aromatic compounds biodegradation in *Sphingopyxis* strains except for DSM 16222). Structure of aromatic compounds (such as benzoate, phenylalanine aminobenzoate, xylene, styrene, and HCH toluene) and their intermediates, and functional genes related to aromatic compounds degradation were shown.

This study has systematically investigated the genetic potential related to aromatic compounds bioremediation of *Sphingopyxis* strains. Phylogenetic analysis revealed that niche specificity had an insignificant influence on the evolutionary relationship. The high percentage of unique and distributed genes in each *Sphingopyxis* strain suggested that *Sphingopyxis* genome had a relatively high plasticity in response to environmental specificity. COG annotation showed that most of the core genes were predicted to be involved in ribosomal structure and biogenesis, amino acid transport, and metabolism, as well as energy production and conversion. Furthermore, COG clustering suggested a possible link between the functional profile and substrate-specific traits. Prediction of the metabolic profile was performed to identify the possible genes associated with aromatic compounds biodegradation (Figure 4). In *Sphingopyxis* strains, most aromatic compounds pathways were involved in benzoate degradation, phenylalanine metabolism, and aminobenzoate degradation. Our study showed that in *Sphingopyxis* strains partial *bph* and *xyl* genes were predicted, and *β*-ketoadipate pathway and peripheral phenylacetyl-CoA pathway were found to be the main pathway of aromatic compounds degradation. In addition, a large number of MGEs were present in the neighborhood of genes related to aromatic compounds metabolisms, which indicated that functional recruitment might be an efficient way to improve the environmental adaptation of bacterial strains in diverse ecosystems.

## Figures and Tables

**Table 1:** Summary for *Sphingopyxis* isolates used in this study.

Stains	Genome size (Mbp)	Staus	No. of chromosomes (plasmid)	No. of 16S operons	G+C (%)	Genome completeness	Isolation source
*Sphingopyxis alaskensis* RB2256	3.37	Complete	1 (1)	1	65.5	93.7%	Surface waters of Resurrection Bay, Alaska
*Sphingopyxis baekryungensis* DSM 16222	3.07	Draft	N/A	2	62.4	94.1%	Seawater of the Yellow Sea, Korea
*Sphingopyxis bauzanensis* DSM 22271	4.26	Draft	N/A	1	63.3	93.2%	Hydrocarbon-contaminated soil
*Sphingopyxis flava* R11H	4.16	Draft	N/A	1	63.8	94.2%	Hexachlorocyclohexane dumpsite
*Sphingopyxis fribergensis* Kp52	5.2	Complete	1 (1)	1	63.8	93.7%	Soil in Freiberg, Saxony, Germany
*Sphingopyxis granuli* NBRC 100800	4.26	Draft	N/A	1	66.4	93.2%	UASB bioreactor
*Sphingopyxis indica* DS15	4.15	Draft	N/A	1	65.7	94.2%	Hexachlorocyclohexane dumpsite in Lucknow
*Sphingopyxis macrogoltabida* 203 N	5.95	Complete	1 (2)	1	64.7	93.7%	Soil
*Sphingopyxis* sp. 113P3	4.66	Complete	1 (1)	1	64.0	93.2%	Activated sludge
*Sphingopyxis* sp. C-1	4.58	Draft	N/A	1	63.7	93.2%	Blooms of cyanobacteria
*Sphingopyxis* sp. EG6	3.88	Complete	1 (1)	1	64.6	93.7%	Industrial cooling water
*Sphingopyxis* sp. FD7	3.94	Complete	1 (1)	1	65.2	93.7%	Industrial cooling water
*Sphingopyxis* sp. MC1	3.65	Draft	N/A	1	65.2	92.8%	Activated sludge
*Sphingopyxis* sp. MG	4.22	Complete	1 (1)	1	66.4	93.2%	Sewage and soil
*Sphingopyxis* sp. MWB1	3.12	Draft	N/A	1	62.8	92.3%	Crude oil contaminated seashore
*Sphingopyxis terrae* subsp. *terrae* 203-1	3.98	Complete	1 (1)	1	64.6	93.2%	Activated sludge
*Sphingopyxis terrae* subsp. *Ummariensis* UI2	3.58	Draft	N/A	1	65.2	93.3%	HCH-contaminated dumpsite
*Sphingopyxis witflariensis* DSM14551	4.31	Draft	N/A	1	63.3	94.6%	Activated sludge
*Sphingopyxis* sp. YF1	4.37	Complete	1 (0)	2	66.6	93.2%	Blooms of cyanobacteria

**Table 2:** Statistics for predicted mobile genetic elements in *Sphingopyxis* genomes.

Stains	IS element	Phage	CRISPR
*Sphingopyxis alaskensis* RB2256	121	2	0
*Sphingopyxis baekryungensis* DSM 16222	167	2	0
*Sphingopyxis bauzanensis* DSM 22271	119	2	0
*Sphingopyxis flava* R11H	196	3	0
*Sphingopyxis fribergensis* Kp52	104	2	0
*Sphingopyxis granuli* NBRC 100800	206	1	1
*Sphingopyxis indica* DS15	186	2	1
*Sphingopyxis macrogoltabida* 203 N	29	14	0
*Sphingopyxis* sp. 113P3	145	7	0
*Sphingopyxis* sp. C-1	35	4	0
*Sphingopyxis* sp. EG6	76	3	0
*Sphingopyxis* sp. FD7	345	8	2
*Sphingopyxis* sp. MC1	122	2	0
*Sphingopyxis* sp. MG	57	4	1
*Sphingopyxis* sp. MWB1	118	1	0
*Sphingopyxis terrae subsp. terrae* 203-1	125	1	0
*Sphingopyxis terrae subsp. Ummariensis* UI2	347	2	0
*Sphingopyxis witflariensis* DSM14551	349	3	2
*Sphingopyxis* sp. YF1	90	2	0

## Data Availability

The datasets used in this study are downloaded from the National Center for Biotechnology Information repository, including both 16S rRNA gene sequences and genomic sequences.

## Supplementary Materials

Supplementary 1 Figure S1: Phylogenetic analysis based on16S rRNA genes of Sphingopyxis strains. Novosphingobium mathurense SM117 was used as an outgroup. Figure S2: Schematic for main gene clusters potentially related to aromatic compounds metabolismsinSphingopyxis strains (except for *S. baekryungensis* DSM 16222).

Supplementary 2 Table S1: Genome-based phylogenetic indicators of Sphingopyxis strains. Table S2: Identification of gene clusters potentially involved in aromatic compounds degradation in *Sphingopyxis* strains. Table S3 Distribution of predicted IS elements in *Sphingopyxis* strains.
