# Supplementary material for: Genome-Wide Analysis Reveals Genetic Potential for Aromatic Compounds Biodegradation of Sphingopyxis
Source: Biomed Res Int. 2020 May 27;2020:5849123. doi: 10.1155/2020/5849123 (PMC7273453; doi:10.1155/2020/5849123)
Supplement: Supplementary 1 — Figure S1: Phylogenetic analysis based on16S rRNA genes of Sphingopyxis strains. Novosphingobium mathurense SM117 was used as an outgroup. Figure S2: Schematic for main gene clusters potentially related to aromatic compounds metabolismsinSphingopyxis strains (except for S. baekryungensis DSM 16222). [file 5849123.f1.docx]

Supplementary Material for

**Genome-wide analysis reveals genetic potential for aromatic compounds biodegradation of *Sphingopyxis***

**Fei Yang****^†^,** **Hai Feng****^†^,** **Isaac Yaw** **Massey, Feiyu Huang,** **Jian Guo, and** **Xian Zhang^*^**

Department of Occupational and Environmental Health, Xiangya School of Public Health, Central South University, Changsha, China

^*^ Correspondence: zixuange2010@126.com

^†^ These authors contributed equally to this work

**Supplementary Figures**


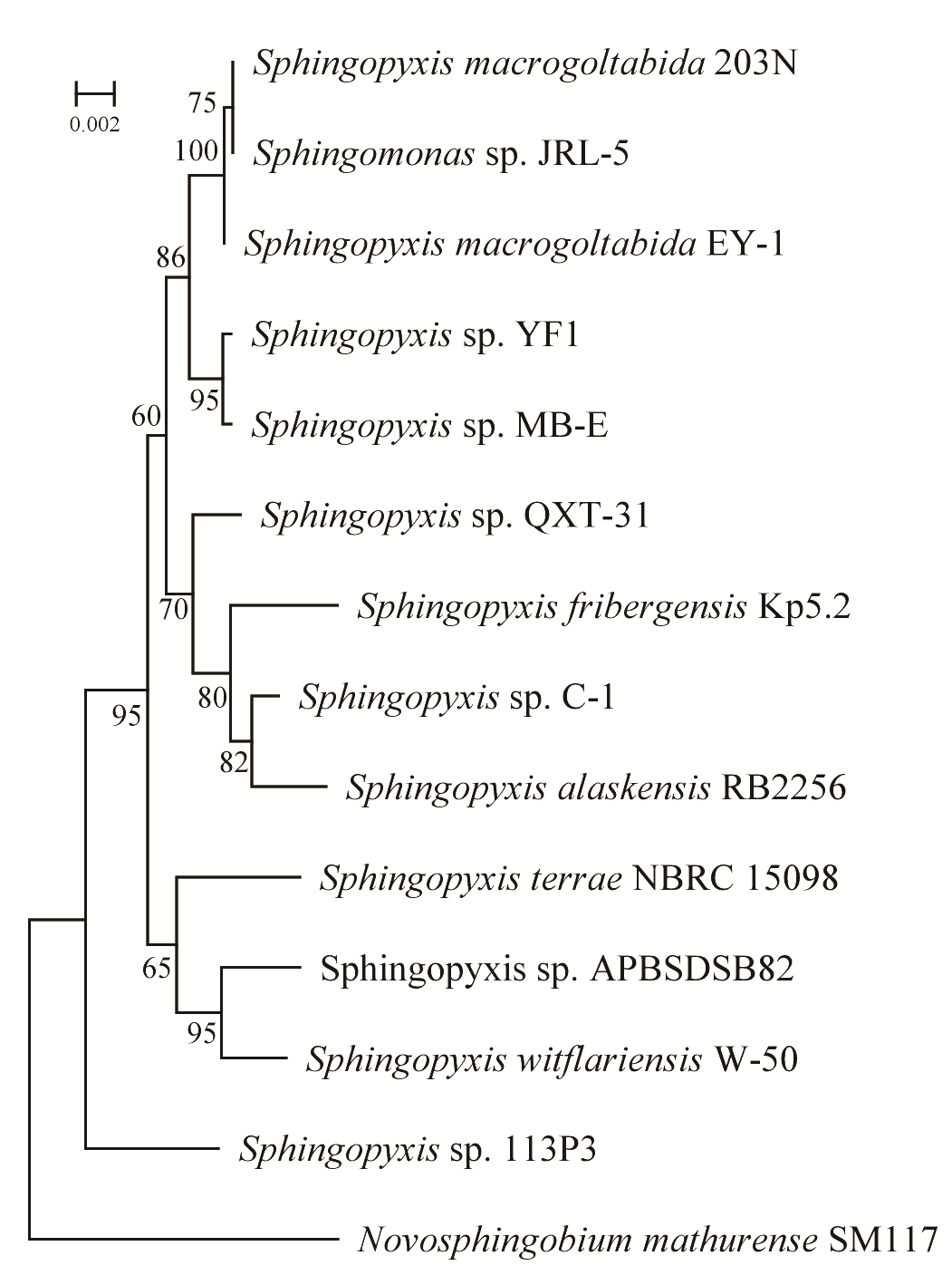


**Figure S1** Phylogenetic analysis based on 16S rRNA genes of *Sphingopyxis* strains. *Novosphingobium mathurense* SM117 was used as an outgroup.


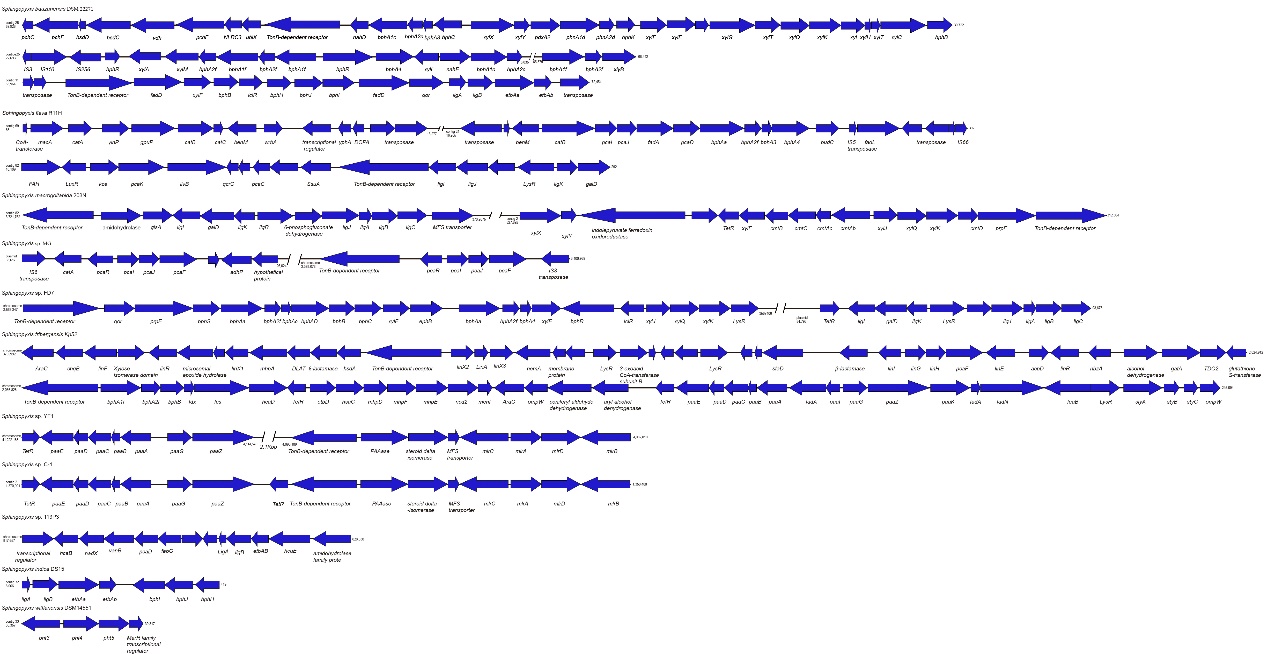


**Figure S2** Schematic for main gene clusters potentially related to aromatic compounds metabolisms in *Sphingopyxis* strains (except for *S. baekryungensis* DSM 16222). ‘//’ represented more than two genes. More details for genes potentially involved in aromatic compounds metabolisms were list in Table S2.
